# The Quality of Interactions Between Staff and Residents With Cognitive Impairment in Nursing Homes

**DOI:** 10.1093/geroni/igaa057.3184

**Published:** 2020-12-16

**Authors:** Anju Paudel, Barbara Resnick, Elizabeth Galik

**Affiliations:** 1 University of Maryland, Glen Burnie, Maryland, United States; 2 University of Maryland School of Nursing, Baltimore, Maryland, United States; 3 University of Maryland School of Nursing, Ellicott City, Maryland, United States

## Abstract

Background: Positive and effective staff–resident interactions are imperative to adequately assess and meet the needs of cognitively impaired residents in nursing homes and optimize their quality of life. Purpose: The purpose of this study was to quantify, describe, and analyze the interaction between staff and cognitively impaired residents in nursing homes, using the Quality of Interaction Schedule (QuIS). Specifically, the following aims were addressed— Aim 1: To quantify and describe the quality of interactions between staff and cognitively impaired residents in nursing homes. Aim 2: To analyze whether the quality of staff–resident interactions vary by resident cognitive status (moderate vs severe) and interaction characteristics (interaction location, interaction situation, interpersonal distance, type of staff, and resident level of participation). Method: This descriptive analysis utilized baseline data from the first 2 cohorts in a randomized clinical trial including 341 residents from 35 nursing homes. Results: Five hundred fifty-six staff–resident interactions were evaluated; majority were positive (n = 466, 83.8%) and the remaining were either neutral (n = 60, 10.8%) or negative (n = 30, 5.4%). The quality of interactions varied by interaction location, interpersonal distance, and resident participation. Conclusion: This study provides some current descriptive information about the quality of staff-resident interactions in nursing homes and the interaction characteristics that might impact these interactions. Future research should focus on decreasing the negative/neutral interactions and explore staff characteristics (e.g., gender, level of experience) and facility factors (e.g., size, ownership) that might influence the quality of interactions.

